# Recent Advances in PROTACs for Drug Targeted Protein Research

**DOI:** 10.3390/ijms231810328

**Published:** 2022-09-07

**Authors:** Tingting Yao, Heng Xiao, Hong Wang, Xiaowei Xu

**Affiliations:** 1 State Key Laboratory of Natural Medicines, Key Lab of Drug Metabolism and Pharmacokinetics, China Pharmaceutical University, Nanjing 210009, China; 2 Guangdong Provincial Key Laboratory of Virology, Institute of Medical Microbiology, Jinan University, Guangzhou 510632, China

**Keywords:** PROTAC, target protein, protein degradation, indication

## Abstract

Proteolysis-targeting chimera (PROTAC) is a heterobifunctional molecule. Typically, PROTAC consists of two terminals which are the ligand of the protein of interest (POI) and the specific ligand of E3 ubiquitin ligase, respectively, via a suitable linker. PROTAC degradation of the target protein is performed through the ubiquitin–proteasome system (UPS). The general process is that PROTAC binds to the target protein and E3 ligase to form a ternary complex and label the target protein with ubiquitination. The ubiquitinated protein is recognized and degraded by the proteasome in the cell. At present, PROTAC, as a new type of drug, has been developed to degrade a variety of cancer target proteins and other disease target proteins, and has shown good curative effects on a variety of diseases. For example, PROTACs targeting AR, BR, BTK, Tau, IRAK4, and other proteins have shown unprecedented clinical efficacy in cancers, neurodegenerative diseases, inflammations, and other fields. Recently, PROTAC has entered a phase of rapid development, opening a new field for biomedical research and development. This paper reviews the various fields of targeted protein degradation by PROTAC in recent years and summarizes and prospects the hot targets and indications of PROTAC.

## 1. Introduction

Targeted protein degradation (TPD) is an emerging therapeutic modality that has the potential to solve the dilemma faced by traditional small molecule targeted therapy. Targeted protein degradation currently mainly degrades target proteins through ubiquitin–proteasome and lysosome. At present, molecular glue and PROTAC technology are the fastest growing in the field of targeted protein degradation [1].

**Figure 1:**
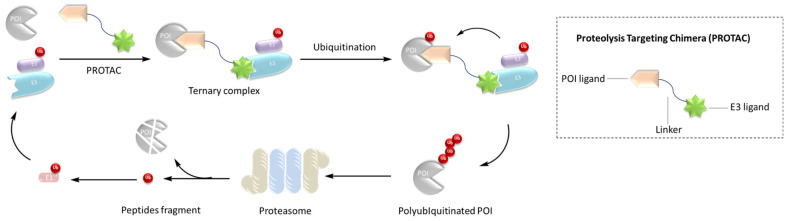
Mechanism of PROTAC.

**Figure 2:**
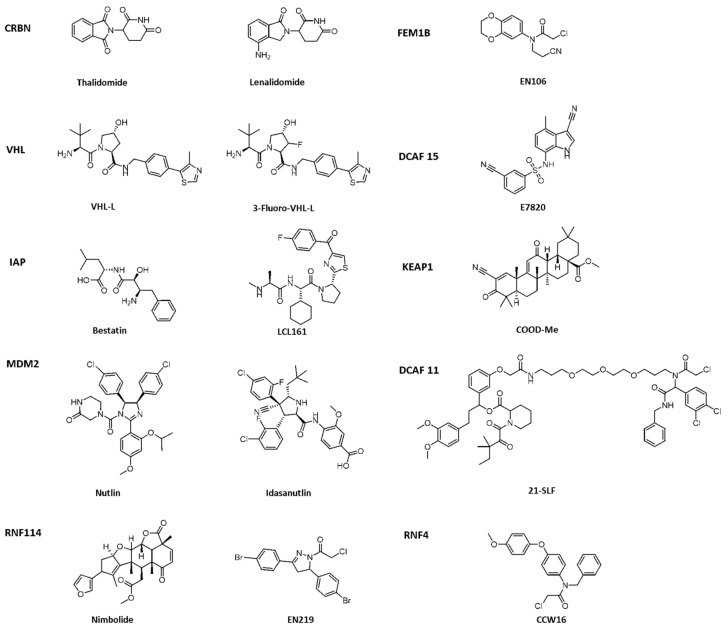
Existing E3 ligands used for targeted protein degradation applications.

Crews et al. introduced PROTAC for the first time in 2001, and PROTAC works by reducing protein levels rather than inhibiting protein function [2,3,4]. As a bifunctional small molecule compound, typically PROTAC consists of two terminals which are the ligand of the target protein and the specific ligand of E3 ubiquitin ligase, respectively, via a suitable linker [5,6,7,8,9,10]. PROTAC degrades target proteins through the ubiquitin–proteasome system (UPS). The general process is that PROTAC binds the target protein (POI) and E3 ligase to form a ternary complex, marking the target protein with the label of ubiquitination. The ubiquitinated proteins are recognized and degraded by the intracellular 26S proteasome [11,12,13,14,15,16,17,18,19] (Figure 1). The E3 ubiquitin ligase has approximately more than 600 members and is the most diverse component of the ubiquitin–proteasome system. The E3 ligases reported in the literature currently used in PROTAC mainly include Cereblon E3 ubiquitin ligase complex (CRBN), Von Hippel–Lindau-containing complex (VHL), inhibitor of apoptosis protein (IAP), and mouse double minute 2 (MDM2). The E3 ligases with the best effect and the highest frequency are mainly CRBN and VHL. Among them, the ligands of CRBN are mainly lenalidomide (Figure 2), thalidomide (Figure 2), and their analogs, while the ligands of VHL are mainly VHL-L (Figure 2) and 3-fluoro-VHL ligand [20,21,22,23,24] (Figure 2). PROTAC structurally connects two ligands through the linkers. The composition and length of the linker play an important role in PROTAC. Generally speaking, the composition and length of the linkers have different effects on degradation activity according to different targets. In addition, linked sites of the linkers also affect degradation activity. The binding sites of POI ligands and E3 ligase ligands are generally in the regions where the ligands are exposed to solvents. The connecting sites are generally connected by amide bonds, carbon atoms, or heteroatoms [25].

Compared with traditional therapies, PROTAC technology has advantages such as wider scope of action, higher activity, and targeting “undruggable” targets. First, PROTAC can degrade the entire target protein to affect protein function, which is expected to solve the potential drug resistance problem faced by current traditional therapies; second, in theory, PROTAC can grasp the target protein through any corner and gap; therefore, PROTAC can target “undruggable target“; third, PROTAC can also affect non-enzymatic functions and expand the drug space of the target. So far, PROTACs have been successfully used to degrade several distinct target proteins associated with all kinds of illnesses, such as cancer, immune disorders, neurodegenerative conditions, cardiovascular diseases as well as viral infections [26,27,28]. In particular, 60 successful cases have demonstrated the effectiveness of PROTACs in degrading target proteins, two of which are currently in clinical trials for prostate and breast cancer treatment [29,30]. PROTAC has emerged as a fresh approach to medication development, providing a fresh method of treating disease. This article reviews the five applications of PROTAC in cancer, immune diseases, neurodegenerative diseases, cardiovascular diseases, and viral infections, summarizes and prospects its potential targets and indications.

## 2. Application of PROTAC in Anticancer

Cancer is one of the worst illnesses threatening human health. In recent years, the treatment of cancer is no longer confined to traditional surgery and radiotherapy and chemotherapy. Targeted therapy and immunotherapy play an important role in anti-cancer treatment. However, there are still no effective targeted drugs for “undruggable targets” such as KRAS and TP53 [31]. The ability to shift the target from “no drug” to “drug” is the most main benefit of PROTAC technology. Traditional targeted drugs need to be firmly bound to the target protein. Since PROTAC protein degrading agent can specifically “label” the target protein only by weakly binding with it, PROTAC degradation agent may solve about 80% of the current “undruggable” proteome. It is a timely help for patients who cannot carry out traditional targeted therapy [32].

### 2.1. Breast Cancer

Breast cancer is the most frequent malignant tumor in women worldwide, and the research and development of new anti-breast cancer drugs has always attracted much attention. The E3 ubiquitin ligase MDM2 ligand nutlin-3 derivative and poly (ADP) ribose polymerase 1 (PARP1) ligand niraparib derivative PROTACs were used to target PARP1 degradation, thus causing human breast cancer cells MDA-MB-231 to undergo apoptosis [33]. Naito Mikihiko et al. reported that an IAP ligand derivative and estrogen receptor α (ERα) ligand 4-hydroxytamoxifen consists of PROTACs linked by alkyl or PEG between the two [34,35]. Their study showed that PROTAC 1 (Table 1) induces ERα degradation, reactive oxygen species (ROS) production, and necrotic cell death in estrogen-dependent breast cancer MCF-7 cells, with therapeutic potential for ERα-positive breast cancer.

The Crews group reported the first VHL-based small molecule PROTAC 2 (Table 1) [36] targeting estrogen receptor-related receptor α (ERRα), which was able to specifically reduce ERRα protein levels in McF-7 cells and MDA-MB-231 tumors at nanomolar concentrations. In addition, they also reported another PROTAC molecule, PROTAC 10 (Table 1), which targets the receptor-interacting serine-threonine kinase 2 (RIPK2). PROTAC 10 can significantly degrade RIPK2 in the acute monocytic leukemia cell line THP-1 cells, which may be an effective option for treating acute monocytic leukemia.

Focal adhesion kinase (Fak) is essential for tumor invasion and metastasis [74,75] and acts as a scaffold for kinases and various signaling proteins [76]. Previously, modulation of Fak activity was limited to kinase inhibitors with a low success rate limited success in clinical studies, so the strategy of PROTAC for FAK degradation was investigated. The Crews group synthesized PROTAC 3, a Fak degrader (Table 1), which significantly exceeded the FAK inhibitor defactinib [37] in Fak signaling and cell migration and invasion in human triple-negative breast cancer (TNBC) cells. Schlaepfer David D et al. synthesized a series of PROTACs [38] based on CRBN ligand and FAK inhibitor PND-1186 through a series of CRBN and PEG connections, among which compound PROTAC 4 (Table 1) observed significant degradation of FAK in human pancreatic cancer cell PA-TU-8988 T cells and PA-TU-8988 T xenograft model in nude mice.

The components of p38 Mitogen-activated protein kinase (p38 MAPK), which includes p38, p381, p382, are tissue-specific and react to cytokines and environmental stress [77,78]. Currently, inhibitors targeting p38α have not shown good efficacy and safety [79,80,81]. The development of selective inhibitors for p38δ is made difficult by the restricted ATP-binding pocket [82,83,84,85]. To cause the degradation of p38 or p38 in MDA-MB-231, the Crews group synthesized PROTAC 5 (Table 1) and PROTAC 6 (Table 1) based on foretinib and VHL, which are p38 and p38 selective degraders [39].

The study by Zhou Daohong et al. [40] showed that the B-cell lymphoma extra-large (BCL-X_L_)-targeting PROTAC molecule PROTAC 7 (Table 1) combined with docetaxel can more effectively inhibit the growth of tumors in MDA-MB-231 breast cancer xenotransplantation model than docetaxel alone, without causing significant changes in body weight. In addition, it has recently been found that the combination of nimbolide, a covalent ligand of RNF114, with BRD4 inhibitor JQ1 to produce PROTAC 8 (Table 1), which targets BRD4, reduced BRD4 expression levels in 231MFP breast cancer cells [41]. Another study presents PROTACs that target the degradation of protein tyrosine kinase 6 (PTK6), consisting of CRBN and VHL E3 ubiquitin ligase ligands and a published PTK6 inhibitor, as a potential treatment for breast cancer [86]. Among them, PTK6 was degraded by PROTAC 9 (Table 1) in MDA-MB231 triple-negative breast cancer cells. Compared with a single PTK6 inhibitor, the compounds showed a better inhibitory effect on ER+ breast cancer cells and platinum-resistant ovarian cancer cells [26].

### 2.2. Hematological Tumors

Hematologic malignancy (HM) is a malignant tumor originating from the hematopoietic system. Clinically, there are three common types of leukemia, multiple myeloma, and malignant lymphoma [87]. Some target proteins in hematological malignancies, such as BCL-X_L_, MDM2, STAT3, and MALT1, are also difficult to be drugged. The emergence of PROTAC technology brings hope for the treatment of such diseases. Herein, PROTAC molecules targeting hematological malignancies in recent years are introduced.

#### 2.2.1. Leukemia

Leukemia is the most prevalent cancerous blood malignancy, and inhibitors targeting leukemia-related proteins are not effective in the treatment of leukemia. PROTAC technology may solve this dilemma.

The most prevalent acute leukemia in adults is acute myeloid leukemia (AML) [88], which is distinguished by aberrant proliferation and weakened differentiation ability of hematopoietic precursor cells, resulting in a large number of immature leukocytes in the bone marrow, peripheral blood, and even other tissues aggregate, while the number of other normal blood cells decreases dramatically [89]. Tripartite motif-containing protein 24 (TRIM24), also known as transcriptional intermediary factor 1α (TIF1α), is a multi-domain protein involved in the transcriptional regulation of the androgen receptor (AR) and other nuclear receptors [90]. A potential prostate cancer therapy target is TRIM24, which has been implicated in various tumorigenesis and disease progression [91,92,93,94,95,96]. Bromodomain inhibitors are far from enough as an anticancer strategy. Bradner’s team synthesized PROTAC 11 (Table 1) based on the TRIM24 bromodomain inhibitor IACS-9571 and the ligand of VHL. PROTAC 11 recruits VHL to cause the effective and selective degradation of TRIM24 in human acute myeloid leukemia MOLM-13 cells [42]. This study proposes TRIM24 as a fresh target for acute myeloid leukemia creation of drugs, providing a new avenue for “undruggable targets”.

MDM2 is an E3 ubiquitin ligase that promotes the degradation of the p53 tumor suppressor gene [97,98]. Wild-type p53 protein is inactivated by overexpressed MDM2 due to its reverse regulation [99]. Therefore, MDM2 is an attractive target for a new therapy for AML specifically for the treatment of MDM2-overexpressing but TP53 wild-type AML. Because p53 stabilization upregulates MDM2 protein levels, which limits the clinical efficacy of MDM2 inhibitors [100], Talpaz Moshe et al. developed an MDM2 PROTAC degrader that binds to and targets MDM2 for degradation, eliminating the inhibition of p53, thereby inducing apoptosis in leukemia cells [101]. Unfortunately, the structure of this compound has not been disclosed. Another team also reported PROTAC 12 (Table 1), a small-molecule degrader targeting MDM2, which effectively induces the rapid degradation of MDM2 in human leukemia cells at a concentration of <1 nm, which can inhibit the growth of B-cell acute lymphoblastic leukemia (B-ALL) RS4-11 cells and human acute myeloid leukemia cell MV4-11 at low nanomolar concentration and induce tumor regression in the RS4-11 xenotransplantation model [43].

FMS-like tyrosine kinase 3 (FLT-3) is a therapeutic target in AML, FLT-3 frequently occurring mutation of an internal tandem duplication (ITD) in the juxta-membrane domain [102,103]. Regarding the limited clinical efficacy of FLT-3 inhibitors [104,105], the Crews group converted the FLT-3 inhibitor quizartinib into the PROTAC molecule PROTAC 13 (Table 1), which induced MV4-11 cells at low nanomolar concentrations and degradation of FLT-3 ITD mutants in MOLM-14 cells. The study showed that PROTAC was able to inhibit cell growth more effectively than the inhibitor alone, and they also demonstrated that the compound was able to induce FLT-3 ITD degradation in vivo. This suggests that the degradation of FLT-3 ITD may provide a useful approach for therapeutic intervention in AML [44].

Signal transducer and activator of transcription 3 (STAT3) is a member of the STAT family, which responds to a variety of cytokines, growth factors, and other signals and activates the expression of downstream genes [106]. Dysregulation of STAT3 contributes to a variety of human cancers as well as many human illnesses. Therefore, STAT3 has long been considered a therapeutic target for diseases such as cancer. Unfortunately, small-molecule drugs targeting STAT3 are difficult to find due to poor specificity and other reasons [107]. In order to solve this problem, Shaomeng Wang’s research group used PROTAC technology to design a small molecule PROTAC 14 (Table 1) that can specifically degrade STAT3 in cancer cells. Apoptosis slows the development of a fraction of acute myeloid leukemia and anaplastic large cell lymphoma cell lines, and at well-tolerated dosages, the chemical completely and permanently eradicates tumors in a number of xenotransplantation mice models. Therefore, a possible cancer treatment method is to degrade the STAT3 protein [45].

The cyclin-dependent kinase (CDK) family are serine/threonine kinases [108] that function in cell cycle regulation and transcription. Excessive activation of CDK proteins results in dysregulated cell proliferation that promotes tumor progression, and inhibition of some CDK family members has been shown to be a viable approach to cancer therapy [109]. But the design of selective small-molecule inhibitors is often hindered by similar ligand-binding pockets. Furthermore, current inhibitors cannot disrupt scaffold function [110]. To solve this problem, Gray’s team used the PROTAC strategy to describe a phthalimide-based degrader, PROTAC 15 (Table 1), since this degrader forms a different ternary complex with the E3 ligase CRBN; so, this degrader is specific and proteome-wide selective for CDK6. PROTAC 15 exploits the selective dependence of AML cells on CDK6 to rapidly degrade CDK6, enabling dynamic mapping of its direct role in coordinating signaling and gene control in AML [46].

BRD2, BRD3, BRD4, and BRDT are the key members of the bromodomain and extraterminal domain (BET) family of proteins, which are crucial for epigenetic control [111,112,113]. A large number of small molecule degraders targeting BRD4 have demonstrated therapeutic activity in preclinical models of AML and inflammatory diseases. In 2015, Bradner’s lab independently reported CRBN-dependent BET protein degradation of PROTAC molecules—thalidomide-based PROTAC 16 (Table 1) [47]. In 2017, Ciulli et al. described a PROTAC molecule PROTAC 17 (Table 1) related to VHL binders [15], the degrader can bind to BRD4 and VHL to mediate the degradation of BRD4. Due to the structural instability of CRBN binder thalidomide, the Rankovic lab discovered new CRBN binders with higher chemical stability and ligand efficiency in 2021 and designed a novel BET PROTAC molecule PROTAC 18 (Table 1) with higher efficiency. Their data showed that PROTAC 18 can inhibit the viability of human acute myeloid leukemia MV4-11 cells at picomolar concentrations (IC_50_ = 3 pM) [48]. Subsequently, Ciulli’s group designed a trivalent PROTAC molecule PROTAC 19 (Table 1) based on MZ1 and the BET inhibitor MT1 that can bind to two BRD4 proteins, which carries two binding domains for BET proteins and one for the E3 binding domain to which ubiquitin ligase binds. They found that PROTAC 19 was 300-fold more active in degrading BRD2 protein than the existing bivalent PROTAC molecules ARV-771 and MZ1. And PROTAC 19 can more effectively inhibit the viability of MV4-11 cells and induce apoptosis of prostate cancer cell line 22RV1 [49].

T-cell acute lymphoblastic leukemia (T-ALL) is a hematological malignancy originating from immature T-cell precursors. A previous study found that T-ALL was dependent on BCL-X_L_ [114]. The BCL-2 protein family member BCL-X_L_ is essential for the survival of cancer cells [115,116,117]. However, BCL-X_L_-specific inhibitors targeted, dose-limiting platelet toxicity, leading to thrombocytopenia, which limits their application in acute leukemia [118]. Konopleva Marina Y investigated the preclinical efficacy of PROTAC7 in T-ALL cell lines in vitro and in living T-ALL patient-derived xenotransplantation (PDX) models. This study shows that T-ALL cells are highly sensitive to PROTAC 7 in vitro. In a living T-ALL PDX model, the use of PROTAC 7 in combination with chemotherapy can alleviate leukemia and prolong patient survival. In conclusion, PROTAC 7 targeting BCL-X_L_ is an efficient and safe adjuvant therapy in T-ALL [119]. In another study, Zheng Guangrong et al. reported two PROTAC BCL-X_L_ degraders, PROTAC 20 (Table 1) [50] and PROTAC 21 (Table 1) [51], which can act in a dose- and time-dependent manner. Degrades BCL-X_L_ in MOLT-4 T-ALL cells with unique selectivity for MOLT-4 cells compared to traditional BCL-X_L_ inhibitors, suggesting that T-ALL can be improved by converting the inhibitor to PROTAC treatment window.

Chronic myelogenous leukemia (CML) is most often caused by the loss of auto-inhibitory constraint of the c-ABL kinase domain in the oncogenic fusion protein BCR-ABL [120,121]. With the advent of BCR-ABL-targeted tyrosine kinase inhibitors (TKIs), CML has become a chronic but manageable disease, but due to the presence of persistent leukemia stem cells (LSCs), all CML patients must receive lifelong treatment [122,123,124]. Therefore, targeting BCR-ABL degradation may bring new benefits to CML patients. In 2017, the Crews group demonstrated PROTACs that bind to VHL and CRBN E3 ligases, respectively, to known kinase inhibitors (such as imatinib, bosutinib, and dasatinib). Western blot showed that the CRBN-based PROTAC molecule PROTAC 22 (Table 1) could degrade the BCR-ABL fusion protein and c-ABL-expressed receptors in K562 CML cells. When using the VHL binder, only dasatinib-based PROTACs observed degradation of c-ABL, and no degradation of BCL-ABL was observed for all VHL-based compounds. No degradation of c-ABL and BCL-ABL was observed with imatinib-based PROTACs [52]. In addition, Naito’s group developed BCR-ABL degraders PROTAC 23 (Table 1) [54] and PROTAC 24 (Table 1) [53] called SNIPER (ABL), which induced the degradation of BCR-ABL protein in BCR-ABL positive CML cells and inhibited the growth of chronic myeloid leukemia K562 cells. Taken together, the above-mentioned multiple studies suggest that the degradation of BCR-ABL is a potential strategy for the treatment of BCR-ABL-driven chronic myelogenous leukemia.

#### 2.2.2. Malignant Lymphoma

New treatment medicines are urgently required to treat T-cell lymphoma (TCL). It was discovered that BCL-X_L_ was essential for the survival of the vast majority of T-cell lymphoma cell lines, patient-derived xenografts, and significant patient samples [125]. Therefore, targeted inhibition of BCL-X_L_ has therapeutic value for some TCL patients. However, targeting BCL-X_L_ small-molecule inhibitors failed due to targeted toxicity leading to thrombocytopenia. To overcome this toxicity, a study explored the therapeutic effect of PROTAC 8 on several TCL cell lines in vitro and in a mouse model with a TCL xenograft. The results show that PROTAC 8’s targeting of BCL-X_L_ preferentially kills BCL-X_L_-dependent TCL cells without causing any obvious platelet damage. In addition, the combination of this degrader and other inhibitors targeting BCL-2 family proteins has broad therapeutic effects on multiple TCL types and other cancer types dependent on BCL-X_L_ [55].

Human mucosa-associated lymphoid tissue protein 1 (MALT1) is a protease and scaffold protein involved in NF-κB signal transduction, which is essential for cell proliferation and survival [126]. Recent studies have found that MALT1 has therapeutic targeting in ABC-type diffuse large B-cell lymphoma (ABC-DLBCL) [127]. Studies have shown that inhibiting the MALT1 protein causes autoimmune disease and death in mice. On the other hand, the degradation of MALT1 protein also has an effective antitumor effect without causing autoimmunity in mice. These findings prompted Ari Melnick’s team to investigate alternative MALT1-targeted treatments for its scaffolding activity. They synthesized many MALT1-targeting PROTACs. The data demonstrated that MALT1 PROTACs induce MALT1 degradation in the human diffuse large B-cell lymphoma cell line OCI-Ly3, suggesting that MALT1 PROTACs may be excellent drugs for the treatment of ABC-DLBCL and other lymphomas [128].

B cell receptor signaling pathway is significantly regulated by Bruton’s tyrosine kinase (BTK). It plays a role in the proliferation, differentiation, and death of B cells and is frequently expressed in several hematological malignancies. Therefore, it is regarded as a key target for the therapy of hematological malignancies. Ibrutinib, its irreversible inhibitor, has transformed the way that patients with chronic lymphocytic leukemia (CLL) and other B-cell cancers are treated, but many patients have developed drug resistance [129,130]. BTK degradation can be used as a strategy to solve the resistance of BTK inhibition [57]. Based on this, Rao Yu’s research group, Zhu Jun’s research group, and Liu Wan Li’s research group worked together to successfully and efficiently degrade a variety of clinically relevant mutant BTK proteins by constructing a novel high solubility BTK protein degradation agent PROTAC 25 (Table 1) and overcomes the clinical resistance of B-cell lymphoma (BCL) to the clinical first-line drug ibrutinib caused by BTK protein mutation. More importantly, the effectiveness of the new strategy to overcome tumor resistance has been validated by in vivo experiments [56]. In addition, Crews’ group also proposed some BTK-degrading PROTAC molecules [57] as potential treatments for patients with ibrutinib-resistant CLL. This article presents VHL-based and CRBN-based PROTACs, compound PROTAC 26 (Table 1) is a PROTAC based on ibrutinib with CRBN-binder. This degrader degrades BTK with a DC_50_ of 6.2 nM and a D_max_ greater than 99% in human Burkitt lymphocyte Namalwa cells. It has better efficacy than ibrutinib in primary cell samples from C481S patients. Subsequently, since the pharmacokinetic properties of PROTAC 26 were not suitable for further in vivo development, they made a series of structural modifications to the linker and E3 recruiting ligands to synthesize the equally effective PROTAC 27 (Table 1), PROTAC 27 was more potent than PROTAC 26, a better pharmacokinetic profile is expected to further explore BTK degradation in vivo [58]. In addition, the research group also designed a BRD4-targeting compound PROTAC 28 (Table 1) in 2015, which demonstrated rapid, efficient, and prolonged BRD4 degradation in all tested Burkitt’s lymphoma (BL) cell lines [59]. Gray’s team developed many compounds based on BTK binders with VHL as well as CRBN binders as recruiting groups for E3 ligases. Among them, the compound PROTAC 29 (Table 1) showed BTK degradation ability in the human AML cell line MOLM-14 and the B-cell lymphoma cell line Ramos B cells, and this substance was reported effective when the QD was 50 mg/kg in a patient-derived xenograft mice model of mantle cell lymphoma (MCL) [60]. Calabrese Matthew F et al. also reported 11 BTK PROTACs linked by PEG chains of different lengths, among which PROTAC 30 (Table 1) was able to degrade BTK [61] with high specificity in both Ramos cells and rats.

The second most prevalent hematological malignancy of multiple myeloma (MM) is a frequent malignant tumor brought on by aberrant clonal plasma cell proliferation. It accounts for 1% of all tumors and 10% of hematological malignancies. CRBN ligand immunomodulatory drugs and proteasome inhibitors are commonly used in the treatment of MM. However, due to the refractory and easy recurrence of MM, the search for new therapeutic strategies is still imminent [131]. Lopez-Girona Antonia et al. described many PROTACs for the treatment of relapsed or refractory multiple myeloma (RRMM), and finally screened PROTAC 31 (Table 1), PROTAC 31 is an Ikaros/Aiolos (IKZF1-3) degrader, which has enhanced antiproliferative and tumoricidal activity in multiple myeloma cell lines, including lenalidomide and pomalidomide resistant cells in vitro, which has strong immune-stimulating activity, currently in phase 2 clinical trials [62]. In addition, Rao Yu’s group used a PROTAC strategy to design and synthesize CDK6-targeted degraders. These PROTACs can effectively and specifically degrade CDK6 at low concentrations. A representative palbociclib-derived PROTAC 32 (Table 1) can strongly inhibit multiple proliferation of myeloma, leukemia, and MCL cells. These findings highlight the value and promise of creating therapeutic compounds based on PROTAC [63].

### 2.3. Colon Cancer

In the United States, colon cancer ranks third among the causes of cancer-related death, and it is considered one of the major cancers that seriously threatens human health, together with lung cancer, prostate cancer, and breast cancer [132]. In recent years, very important progress has occurred in the field of treatment of this common disease, targeting relevant pathogenic proteins for degradation, such as ERK, CDK9, BRD4, TRK, etc., and has been shown to be effective. A recent study demonstrated that a covalent inhibitor-based PROTAC 33 (Table 1) targeting extracellular-regulated kinase 1-2 (ERK1-2) could target malignant melanoma cells ERK1-2 degrades and inhibits the phosphorylated ERK1-2 signaling pathway in A375 and human colon cancer cells HCT116, and it is experimentally confirmed that the binding to CRBN is the key to the degradation of ERK1-2 [64].

The first example of a PROTAC that selectively degrades CDK9 was reported by Rana Sandeep et al., who found that PROTAC 34 (Table 1) degraded CDK9 in a dose-dependent manner in human colorectal cancer cell line HCT116 cells. While the levels of CDK2 and CDK5 in cells remained unchanged, indicating that the compound selectively degrades CDK9, this study suggests that strategies targeting CDK9 degradation may be useful in the treatment of colon cancer [65]. In 2019, the Crews team published a study targeting BRD4 degradation, which showed that a nutin-based PROTAC, PROTAC 35 (Table 1), was able to degrade the human colon cancer cell line HCT116 cells at nanomolar concentrations. They also found that PROTAC 35 inhibited the proliferation of many wild-type p53 cancer cell lines more effectively than PROTACs that degraded BRD4 using VHL [66].

Liu Jing et al. reported two degraders of tropomyosin receptor kinase (TRK), PROTAC 36 and 37 (Table 1), the compound prepared in this paper is composed of CRBN E3 ligase binding agent, which is connected with TRK inhibitor [133] as the target part of TRK. In KM12 colon cancer cells, it was discovered that these two substances decreased the levels of the TPM3-TRKA fusion protein. PROTAC 37 also decreased the levels of the AGBL4-TRKB and ETV6-TRKC fusion proteins. Furthermore, both CRBN-based PROTACs showed favorable plasma exposure levels in mice, thus, these two compounds are valuable chemical tools to study the in vivo function of TRK fusions during colon carcinogenesis [67].

The most cancer-related fatalities occur from lung cancer worldwide, 85% of which are caused by non-small-cell lung cancer (NSCLC). In the past two decades, significant progress has been made in the treatment of NSCLC [134] and targeting pathogenic targets for degradation through the PROTAC strategy has created new opportunities for the treatment of NSCLC as a new therapeutic approach. Hwang’s team designed and synthesized several ALK-PROTAC molecules to degrade anaplastic lymphoma kinase (ALK) fusion proteins. One of the compounds, PROTAC 38 (Table 1), effectively induced ALK degradation and inhibited the growth of the ALK fusion-positive cell line SU-DHL-1 and the human NSCLC cell line H3122, and also inhibited tumor growth in the H3122 xenograft model [68]. Furthermore, in 2018, Gray Nathanael S et al. disclosed the development of the first PROTACs targeting ALK degradation. These PROTACs were based on the binding of ALK inhibitors to E3 ubiquitin ligase ligands, and they screened two compounds, PROTAC 39 (Table 1) and 40 (Table 1). These two compounds efficiently induced the degradation of ALK in NSCLC, anaplastic large cell lymphoma (ALCL), and neuroblastoma (NB) cell lines. As potential chemotherapeutics for NSCLC, their data also suggest that compounds targeting ALK degradation also promote the degradation of other kinases, such as PTK2, Aurora A, FER, and RSK1 for further investigation [69]. In the same year, a successful example of targeting EGFR and PARP dual PROTACs was introduced for the first time in an article by Li Hua et al., in which novel dual PROTACs were synthesized using gefitinib, olaparib, CRBN, or VHL E3 ligands as substrates. Among them, compound PROTAC 41 (Table 1) successfully degraded both EGFR and PARP in H1299 human NSCLC cells. This research will substantially expand the PROTAC method’s application possibilities and create a new avenue for the development of human non-small cell lung cancer drugs [70].

Macrophage migration inhibitory factor (MIF) is involved in diseases through protein-protein interactions in cancers and inflammations such as melanoma [135], neuroblastoma [136], and lung cancer [137]. Studies have found that MIF is down-regulated by protein degradation. Expressing [138] or reducing the level of MIF [139,140] can reduce tumor metastasis and induce antitumor responses [141]. Therefore, MIF may be a new target for cancer therapy. Dekker Frank J’s team reported the first MIF-targeting PROTAC molecule PROTAC 42 (Table 1) in 2021. Their research data showed that this degradation agent can inhibit the proliferation of A549 cells by downregulating the level of MIF in non-small cell lung cancer A549 cells and inhibiting MIF-related signal transduction, proving the potential of PROTAC technology in the treatment of NSCLC [71].

### 2.4. Prostatic Cancer

Prostate cancer is the second most prevalent cancer to cause mortality in males and has a significant impact on men’s health globally [142]. Scientists have never stopped exploring treatment strategies for prostate cancer. Naito’s team developed PROTAC 43 (Table 1), which showed potent protein-degrading activity against AR. In addition, PROTAC 43 potently induced caspase activation and apoptosis in prostate cancer cells compared to AR inhibitors. These findings imply results suggest that targeting AR degradation may be one of the treatment approaches for AR-dependent proliferation in prostate cancer [72].

### 2.5. Pancreatic Cancer

Pancreatic cancer is one of the deadliest malignancies to us. Pancreatic cancer survival rates have remained largely unchanged since the 1960s [143]. The research on pancreatic cancer has never stopped, and by causing the degradation of associated kinase proteins, it may be a possible method for the therapy of pancreatic cancer. The IKK protein kinase family includes TANK-binding kinase 1 (TBK1), a key player in innate immunity. Some studies have found that abnormal TBK1 can cause a variety of diseases and inhibiting the activity of TBK1 can slow or prevent the growth of cancer cells. Therefore, TBK1 is a crucial target for research in the process of tumorigenesis, but there is still no approved TBK1 inhibitor [144]. However, the Crews group reported a PROTAC that induces TBK1 degradation, and PROTAC 44 (Table 1) [73] may serve as an alternative strategy. Interestingly, the Kd of this compound binding to TBK1 in mouse pancreatic cancer Pan02.13 cells was 4 nM. After 16 h of treatment, DC_50_ was 32 nM and D_max_ was 96%. In addition, this compound could not degrade the TBK1 homologous protein IKKɛ with a binding affinity of 70 nM Kd.

## 3. Application of PROTAC in Immune Diseases

Immune inflammatory diseases are very common diseases in life, such as rheumatoid arthritis, systemic lupus erythematosus, ulcerative colitis, and so on. These diseases are threatening people’s health all the time. Scientists’ research on the treatment strategies for these diseases has never stopped. As an emerging strategy, PROTAC has penetrated into the treatment field of immune-inflammatory diseases. The following introduces the use of PROTAC in several applications in immune inflammatory disease targets.

### 3.1. IRAK3

Interleukin-1 receptor-associated kinase 3 (IRAK3) is a member of the IRAK family [145], and related studies have shown that IRAK3 can inhibit pro-inflammatory signaling in congenital leukocytes [146]. In addition, knockout of the IRAK3 gene in mouse bone marrow cells promotes the proliferation of effector T cells, which is conducive to enhancing the host response to checkpoint inhibition and overcoming immunosuppression [147,148]. Therefore, IRAK3 is a potential target for immune diseases. Edmondson’s team first released the IRAK3 PROTAC molecule PROTAC 45 (Table 2) [149] based on the linkage of the IRAK3 ligand and CRBN ligand. Their data showed that more than 98% of IRAK3 was degraded in human monocytic leukemia THP1 cells and primary macrophages. This study provides a good tool for IRAK3 degradation.

### 3.2. IRAK4

Interleukin-1 receptor-associated kinase 4 (IRAK4) is a key protein in the immune response mediated by toll-like receptors and interleukin receptors and has been identified as an autoimmune disease and cancer dual target [133,150,162,163]. At present, the inhibitor [164] against IRAK4 has not yet been approved. Therefore, using PROTAC technology to knock out IRAK4 may be an alternative strategy for the treatment of IRAK4-related diseases. Anderson Niall A. et al. developed and designed some PROTACs [150] that induce the degradation of IRAK4, among which the compound PROTAC 46 (Table 2) successfully induced the degradation of IRAK4 in peripheral blood mononuclear cells (PBMC) and human dermal fibroblasts. Dai Xuedong et al. also reported an IRAK4-targeting degrader, PROTAC 47 (Table 2) [151], and their data showed that more than 90% of IRAK4 was rapidly degraded after treating HEK293T cells with this compound for 24 h. In another study, Duan Wenhu et al. [152] also described and screened a compound targeting IRAK4, PROTAC 48 (Table 2), which induces IRAK4 degradation in diffuse large B-cell lymphoma OCILY10 and TMD8 cells. Meanwhile, PROTAC 48 suppressed the proliferation of cell lines expressing the B-cell lymphoma MYD88 L265P mutant and hindered the IRAK4-NF-B signaling pathway. In addition, KT-474, a potential “first-in-class” IRAK4 oral protein degrader developed by Kymera, has entered phase 1 clinical trials. The trial results showed that, in healthy volunteers, a single dose of KT-474 dose-dependently reduced the levels of IRAK4 and various pro-inflammatory cytokines with good safety and tolerability. These studies illustrate the potential application of IRAK4 degraders for the management of oncology and inflammatory indications.

### 3.3. HDAc3

A class of proteases known as histone deacetylases (HDAcs) are responsible for altering chromosomal shape and controlling gene expression. Inflammatory illnesses such as asthma and chronic obstructive pulmonary disease are affected by HDAc3, which is crucial [165]. Dekker’s group [153] synthesized PROTAC 49 (Table 2) for the degradation of HDAc3. PROTAC49 is a linker between the HDAc inhibitor anthranilide derivative and the CRBN ligand pomalidomide. Their results showed that PROTAC49 had a small impact on gene expression in RAW 264.7 macrophages activated by lipopolysaccharide/interferon, and was able to selectively downregulate HDAc3 levels compared to biochemical evidence using siRNA. That same year, Liao’s team [154] developed PROTAC 50 (Table 2), the compound PROTAC 50 induces selective and efficient degradation of HDAC3. The dose-limiting toxicity of traditional HDAC inhibitors may be overcome by PROTAC 50’s catalytic mechanism of action and isoenzyme selectivity.

### 3.4. HDAc6

Studies have shown that HDAC6 is indispensable for the assembly and activation of the NLRP3 inflammasome. Furthermore, activation of the NLRP3 inflammasome is primarily dependent on the zinc-finger ubiquitin-binding domain of HDAC6 rather than its deacetylation function [166,167]. Therefore, traditional small-molecule inhibitors targeting the deacetylation domain of HDAC6 are not suitable for inhibiting the activation of the NLRP3 inflammasome. In 2021, He’s group reported an HDAC6 degrader PROTAC 51 (Table 2) based on the natural product indirubin derivatives and the CRBN ligand pomalidomide. Their data showed that compound PROTAC 51 can downregulate NLRP3 levels in THP-1 cells that construct the NLRP3 inflammasome activation model, accompanied by downregulation of related cytokines such as IL-1β. This study sheds light on the value of targeted protein degradation strategies in the treatment of inflammatory disorders to some extent [155].

### 3.5. HPGDs

Excessive hematopoietic prostaglandin synthase 2 (PGD2) causes many diseases such as allergic disease, physiologic sleep, and Duchenne muscular dystrophy [168]. PGD2 synthesis requires the participation of hematopoietic prostaglandin D synthases (H-PGDs); therefore, H-PGDs are possible treatment targets for such diseases, and in vivo research has also shown that inhibition of H-PGDs is available in the management of allergic inflammation [169]. It is vitally necessary to develop novel therapeutic methods that specifically target H-PGDs as the inhibitors for inhibiting H-PGDs that have been produced so far are not therapeutically effective. In 2021, Demizu et al. [156] reported PROTAC 52 (Table 2) for targeting H-PGDs for degradation and sustained inhibition of PGD2 production. Their findings suggest that knockdown of H-PGDs protein through the PROTAC strategy, thereby inhibiting the production of PGD2, has the potential to become a new therapeutic modality.

### 3.6. IDo1

Indoleamine 2,3-dioxygenase 1 (IDO1) is an enzyme that causes immunological tolerance by preventing T cells from proliferating [170]. Recent studies have found that IDO1 plays an important role in cancer immune escape [171]. In a paper, the Xie team published the first PROTAC 53 (Table 2) that induces downregulation of IDO1 protein levels, a degrader that induced more than 93% of IDO1 protein to be degraded by UPS in HeLa cells [157]. The study not only demonstrates the feasibility of degrading IDO1 but also provides a method to investigate the role of IDO1 protein in tumor immune evasion.

### 3.7. Sirt2

The sirtuins family includes sirtuin 2 (Sirt2), by deacetylating a variety of substrates, it participates in various biological processes including gene silencing, cell cycle regulation, metabolism, and apoptosis. Sirt2 dysregulation has been linked to cancer development, type II diabetes, bacterial infections, and neurological disorders [172,173,174,175,176,177]. This suggests that Sirt2 may be a viable drug intervention target. Jung Manfred et al. reported for the first time the compound PROTAC 54 (Table 2) [158] used to induce Sirt2 degradation, which is composed of Sirt2 inhibitor and CRBN ligand. The results showed that PROTAC 54 can selectively and dose-dependently degrade sirt2 compared with Sirt2 inhibition. Subsequently, they designed and developed a Sirt2 degrader PROTAC 55 (Table 2) [159] with a more optimized performance compared with PROTAC 54. PROTAC 55 can degrade Sirt2 at a 10-fold lower concentration than PROTAC 54.

### 3.8. PCAF/GCN5

A set of epigenetic proteins called P300/CBP-associated factor (PCAF) and general control nonderepressible 5 (GCN5) are essential for a number of cellular processes, such as DNA damage repair, metabolic control, and cell proliferation and differentiation [178]. However, chemical inhibition targeting the PCAF/GCN5 bromodomain was not sufficient to attenuate the inflammatory response of PCAF-deficient immune cells. In 2018, Tough David F et al. first reported a compound [160] targeting PCAF/GCN5 degradation, PROTAC 56 (Table 2), which induced PCAF/GCN5 degradation in THP1 cells at nanomolar concentrations. Meanwhile, the decline in PCAF/GCN5 inhibited the production of many inflammatory factors, which further illustrates the importance of constructing a new PROTAC strategy to prevent inflammation.

### 3.9. RIPk2

The receptor-interacting protein kinase (RIPK) family consists of RIPK1, RIPK2, and RIPK3, which play important roles in inflammation and innate immunity [179]. Numerous inflammatory cytokines are released when RIPK2 is activated, and dysregulation of this pathway is closely related to autoimmune disorders including inflammatory bowel disease. Despite both RIPK1 and RIPK2 inhibitors having entered clinical studies, RIPK inhibitor medications have not yet received approval. The Crews team announced a RIPK2 degrader PROTAC 57 (Table 2) [36] based on the RIPK2 binder and VHL binder in 2015. Their research data showed that PROTAC 57 can degrade more than 95% of RIPK2 at nanomolar concentrations. In 2020, Harling’s team synthesized RIPK2 degraders PROTAC 58 (Table 2) and 59 (Table 2) [161] based on the IAP binder and CRBN binder. They found that compared with PROTAC 57, The effect of PROTAC 58 and 59 in degrading RIPK2 in THP-1 cells is not satisfactory. Although PROTAC 57 has a strong degradation ability, the study found that its binding ability to RIPK2 is much weaker than that of PROTAC 58 and 59.

### 3.10. ASK1

Apoptosis signal-regulating kinase 1 (ASK1) is a widely expressed protein kinase that is extremely redox-sensitive and is directly involved in the regulation of apoptosis and signaling pathways such as inflammation and fibrosis under oxidative stress [180]. Previous animal-level studies have shown that inhibition of ASK1 can effectively reduce liver and kidney injury and fibrosis and is expected to be a possible target for the therapy of nonalcoholic steatohepatitis (NASH) [181] and diabetic kidney disease (DKD) [182] and other diseases. At present, the development of ASK1 inhibitor GS4997 has entered phase III clinical trials [183], but its two key clinical trials for the treatment of NASH have failed one after another, casting a shadow over the development of drugs for this target. PROTAC technology can achieve low-dose degradation of target proteins. Therefore, targeting ASK1 degradation through PROTAC molecules may develop into a new approach to the treatment of diseases such as NASH and DKD.

## 4. Application of PROTAC in Neurodegenerative Diseases

Neurodegenerative diseases are an area in need of new therapies and molecular insights. The aggregation of misfolded proteins such as tau protein and α-synuclein protein is the main cause of such diseases, and they cannot be modulated by traditional small molecule drugs; therefore, the treatment of neurodegenerative diseases has always been a challenge. In recent years, the use of PROTAC technology to degrade target proteins has become a new treatment method. Therefore, PROTAC technology is expected to play a potential role in neurodegenerative diseases caused by protein aggregation.

### 4.1. Alzheimer’s Disease

Tau protein is a microtubule-associated protein abundant in neurons and plays a role in axonal transport and microtubule stabilization [184]. Abnormal regulation of tau protein is the cause of numerous neurodegenerative disorders such as Alzheimer’s disease (AD). Therefore, tau is a possible therapeutic target for neurodegenerative disorders. In 2016 and 2018, Li Yan-Mei and Jiang Zhengyu et al. revealed peptide-based induction of tau degradation in PROTACs respectively [185,186]. In 2019, Kargbo’s group reported the first small-molecule tau degrader [187]. In this paper, six tau-targeting PROTACs were developed based on CRBN and VHL binders. The results showed that tau-targeting PROTACs successfully degraded tau in human tau-p301L and tau-a152T neurons, and several favorable pharmacokinetic parameters were shown. In the same year, Haggarty’s team also announced the synthesis of a series of novel targeting tau PROTACs [188]. The representative compound PROTAC 60 (Table 3) could effectively degrade wild-type and mutant tau. Furthermore, PROTAC 60 preferentially degrades tau in FTD neurons of frontotemporal dementia compared to normal cells. In 2021, Wang Jian-Zhi et al. synthesized a PROTAC based on the binding of tau ligand and VHL ligand, named PROTAC 61 (Table 3) [189]. They used PROTAC 61 in both in vitro and in vivo investigations. The experimental results indicated that PROTAC 61 effectively induced tau degradation under both physiological and pathological conditions. At the same time, they demonstrated that knocking out tau did not cause significant abnormalities in mice. These data suggest that induction of tau protein degradation using PROTAC technology is a possible method for treating neurodegenerative illnesses including AD.

Glycogen synthase kinase 3 (GSK-3) is a class of serine/threonine protein kinases [194]. Relevant studies have demonstrated that GSK-3β can boost the phosphorylation of tau protein and the production of the amyloid-β peptide to induce AD. And GSK-3β has a strong pro-inflammatory effect leading to neuronal loss [195,196,197]. Therefore, GSK-3β is thought to be a potential target for neurodegenerative disorders. Sun Haopeng et al. developed many bifunctional PROTACs targeting GSK-3β for the first time [190]. They can achieve nanomolar degradation of GSK-3β. Among them, the representative compound PROTAC 62 (Table 3) can degrade more than 44% of GSK-3β; additionally, it is worth noting that PROTAC 62 can also prevent the death of mouse hippocampal neurons HT-22 cells induced by glutamate. Their study is of great significance, which presents a fresh approach to the creation of GSK-3β degraders.

### 4.2. Huntington’s Disease

An inherited neurological disorder called Huntington’s disease (HD) is brought on by the HTT gene’s exon 1 developing more than 35 CAG repeats, and the resulting mutant huntingtin (mHtt) accumulates in nerve cells [198]. These aggregates can cause nerve cells to die, which can lead to numerous symptoms, including motor impairment and cognitive deficits. In 2017, Ishikawa’s group [191] designed PROTACs 63 (Table 3) and 64 (Table 3) against this target. PROTAC 63 and PROTAC 64 successfully induced downregulation of mHtt protein levels in HD patient primary cells and mHtt-transfected HeLa cells. Subsequently, they synthesized a new PROTAC 65 (Table 3) [192]. The newly synthesized PROTAC was synthesized with IAP inhibitor MV1 and mHtt ligand through PEG linkage, and the newly synthesized compound showed stronger affinity compared with PROTAC 63 and PROTAC 64. Furthermore, the newly synthesized PROTAC was able to degrade mHtt in fibroblasts from HD patients in a time- and dose-dependent manner.

### 4.3. Parkinson’s Disease

Parkinson’s disease is a motor system-affecting, progressive neurodegenerative condition, and its main feature is the neuronal cytoplasmic aggregation of Lewy bodies composed of aggregates of α-synuclein protein, leading to neuronal degeneration [199]. In 2020, a PROTAC that induces α-synuclein protein degradation was reported by Kargbo’s group [193]. Six key PROTACs were screened in the paper. These six compounds can target α-synuclein protein degradation, and the representative PROTAC 66 (Table 3) can significantly reduce the protein level of α-synuclein in HEK293 TREX u-syn A53T cells, the D_max_ value is 65%. It can be seen that this compound can be utilized as a possible Parkinson’s disease medication and has broad prospects.

## 5. Application of PROTAC in Cardiovascular Diseases

3-hydroxy-3-methylglutaryl coenzyme A reductase (HMGCR) catalyzes 3-hydroxy-3-methylglutaryl coenzyme A in the cholesterol synthesis pathway. HMGCR is a target of statins for the prevention and treatment of cardiovascular diseases [200,201]. In 2020, Luo’s team reported a series of PROTAC molecules [202], among which PROTAC 67 (Table 4) has the greatest impact on the HMGCR protein’s ability to degrade in Chinese hamster ovary SRD15 cells. Additionally, PROTAC 67 activates the sterol regulatory element-binding protein pathway (SREBP) and blocks cholesterol synthesis. That same year, Xiang’s group [203] reported two kinds of lovastatin acid and VHL ligand-conjugated HMGCR targeting PROTAC 68 (Table 4) and 69 (Table 4), and PROTAC 68 could effectively degrade HMGCR in HepG2 cells (DC_50_ = 120 nM). In vivo studies have shown that PROTAC 69 induces HMGCR breakdown and cholesterol reduction in mice with diet-induced hypercholesterolemia.

## 6. Application of PROTAC in Antiviral

Infection with hepatitis C virus (HCV) is the main cause of chronic liver disease, in which the hepatitis C virus (HCV) NS3 protein plays an important role [204,205]. Although VX-950, an inhibitor of the NS3/4A protease, has been authorized for the treatment of HCV, patients are prone to develop drug resistance, so a new treatment method is urgently needed to solve this problem. Yang Priscilla L. et al. synthesized many NS3-targeting PROTACs [206] by linking VX-950 and CRBN Binder based on the PROTAC strategy. Among them, the representative compound PROTAC 70 (Table 5) can effectively degrade NS3 in human hepatoma adherent Huh7.5 cells. In addition, PROTAC 70 can also degrade V55A and A156S mutant NS3. Therefore, the successful discovery of this degradation agent is a boon for HCV-infected patients. This study also suggests that PROTACs may also be potential antiviral drugs, a strategy that has also been used to target SARS-CoV-2 as it emerges. The major proteases (Mpro and PLpro) [207] and RNA-dependent RNA polymerase (RdRP) [208] of SARS-CoV-2 are currently targeted by small molecule inhibitors [209]. They could be potential targets for PROTAC molecules.

## 7. Conclusions and Prospects

PROTAC technology has been developed for 20 years, and some molecules have entered the clinical stage; this sheds light on the huge therapeutic potential of PROTAC in tumors, immune diseases, neurodegenerative diseases, cardiovascular diseases, and viral infections. First, targets for drug resistance were particularly sensitive to PROTACs. The mainstay of cancer treatment in the past has been chemotherapy, but acquired resistance to chemotherapeutic drugs hinders clinical application, resulting in disease recurrence. The subsequent development of kinase inhibitors and immunotherapy have also exposed the problem of drug resistance. Since PROTACs affect protein function, including enzymatic and non-enzymatic functions, by clearing the entire target protein, this technology is expected to address potential drug resistance faced by current treatments. The second is that PROTACs may aim for “undruggable targets.” The majority of small-molecule medications or large-molecule antibodies need the active site of a binding enzyme or receptor to work; however, it is thought that over 80% of proteins in human cells don’t have these sites. PROTACs, on the other hand, can grab target proteins through any nooks and crannies. Third, PROTACs can affect non-enzymatic functions. Conventional small-molecule medications often work by stopping their targets’ enzyme activity. Accumulating studies have shown that PROTACs have the potential to increase the “druggable space” of targets and to regulate protease as well as non-enzyme functions. Some of the difficulties posed by conventional small molecule inhibitors can be overcome. Of course, in terms of clinical practice, PROTAC drugs are still in a relatively early stage, and there are challenges such as slow development of PROTACs and a slow success rate, poor membrane permeability and oral bioavailability, and insufficient evidence from human clinical studies. However, with the accumulation of time and in-depth research, these problems will basically be solved, and once a clinical breakthrough is formed, it will open a new era of drug innovation. PROTAC has a wide range of targets and a huge market. It is believed that with the continuous progress and improvement of this technology, PROTAC can become as successful as small molecule inhibitors, monoclonal antibodies, and immunotherapy, so that more patients with diseases can benefit from it.

## Figures and Tables

**Table 1:** Representative PROTACs for cancer.

Indication	PROTAC	Target	Structure	Activity		Ref.
				DC_50_	D_max_%	
Breast cancer	1	ERα	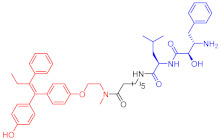	-	-	[34]
	2	ERRα	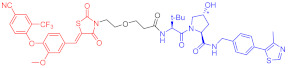	100 nM	86	[36]
	3	FAK	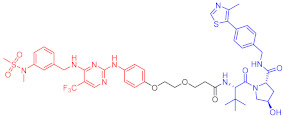	3 nM	99	[37]
	4	FAK	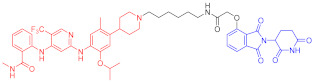	-	>90	[38]
	5	p38α	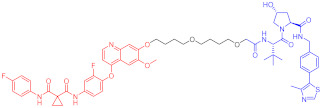	7.16 nM	97.4	[39]
	6	p38δ	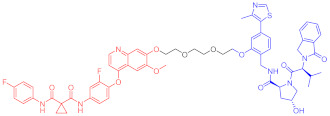	46 nM	99.4	[39]
	7	BCL-X_L_	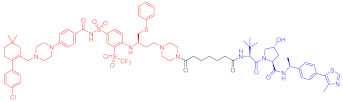	63 nM	90.8	[40]
	8	BRD4	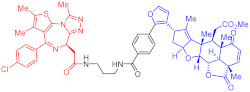	-	-	[41]
	9	PTK6	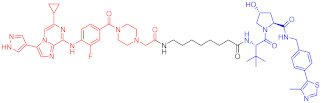	-	-	[26]
AML	10	RIPK2	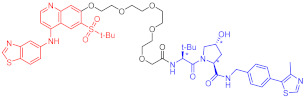	1.4 nM	>95	[36]
	11	TRIM24	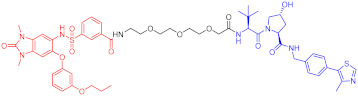	-	-	[42]
	12	MDM2	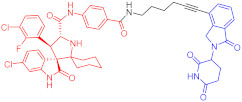	1.5 nM	-	[43]
	13	FLT-3	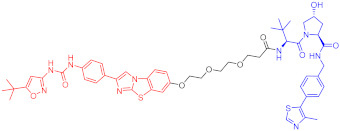	-	-	[44]
	14	STAT3	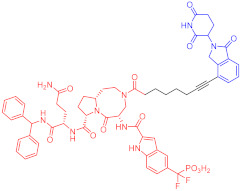	-	>90	[45]
	15	CDK6	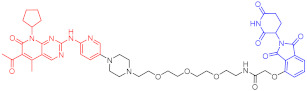	-	-	[46]
	16	BRD4	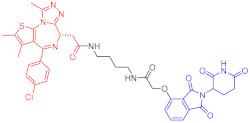	430 nM	-	[47]
	17	BRD4	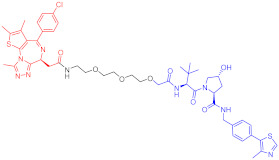	-	-	[15]
	18	BRD4	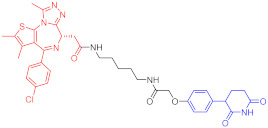	<1 nM	99	[48]
	19	BRD4	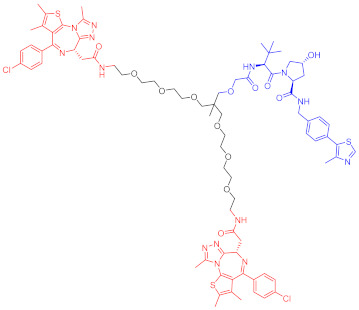	-	-	[49]
T-ALL	20	BCL-X_L_	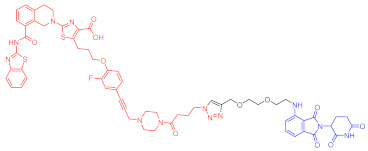	50 nM	>85	[50]
	21	BCL-X_L_	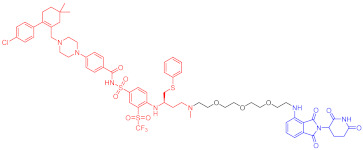	2.5 nM	-	[51]
CML	22	BCR-ABL	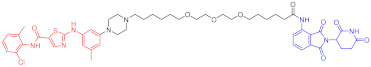	-	>80	[52]
	23	BCR-ABL	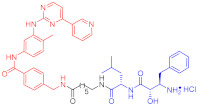	-	-	[53]
	24	BCR-ABL	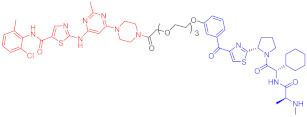	-	-	[54]
TCL	8	BCL-X_L_	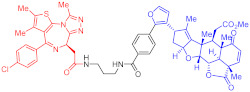	-	-	[55]
BCL	25	BTK	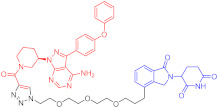	29 nM	-	[56]
	26	BTK	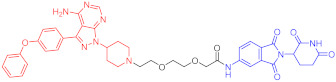	6.2 nM	99	[57]
	27	BTK	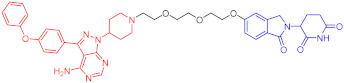	7.9 nM	95	[58]
	28	BTK	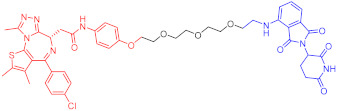	<1 nM	-	[59]
	29	BTK	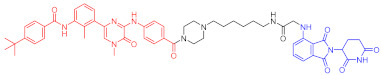	-	-	[60]
	30	BTK	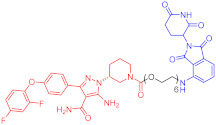	5.9 nM	-	[61]
MM	31	IKZF1-3	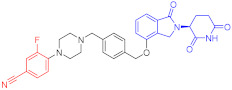	-	-	[62]
	32	CDK6	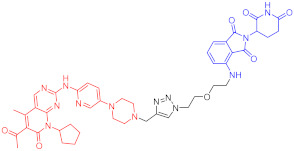	8.6 nM	-	[63]
Colon cancer	33	ERK1-2	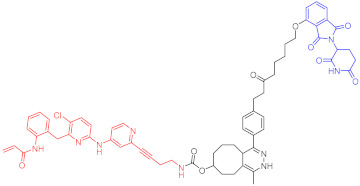	-	-	[64]
	34	CDK9	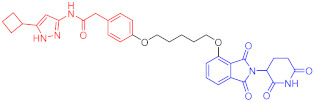	-	-	[65]
	35	BRD4	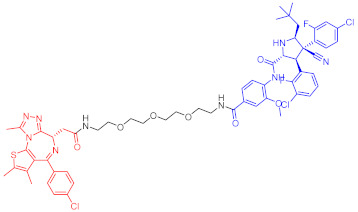	32 nM	98	[66]
	36	TRK	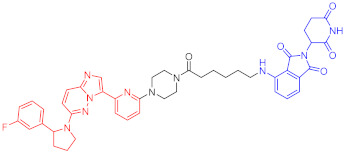	0.48 nM	-	[67]
	37	TRK	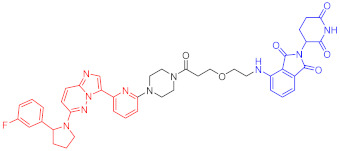	0.36 nM	-	[67]
NSCLC	38	ALK	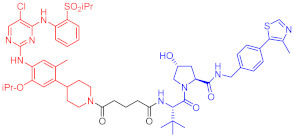	-	-	[68]
	39	ALK	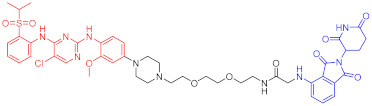	50 nM	-	[69]
	40	ALK	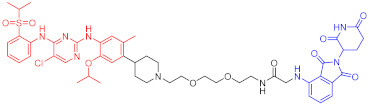	50 nM	-	[69]
	41	EGFR; PARP	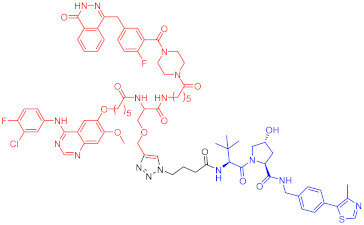	0.47 μM	-	[70]
	42	MIF	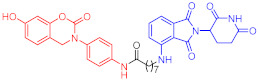	100 nM	>90	[71]
Prostatic cancer	43	AR	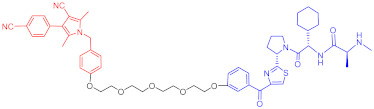	-	-	[72]
Pancreatic cancer	44	TBK1	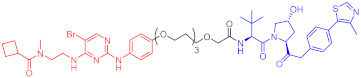	32 nM	96	[73]

Note: red: molecule to bind to POI, black: linker, blue: ligand of E3 ligase.

**Table 2:** Representative PROTACs for immune diseases.

Protac	Target	Structure	Activity		Ref.
			DC_50_	D_max_%	
45	IRAK3	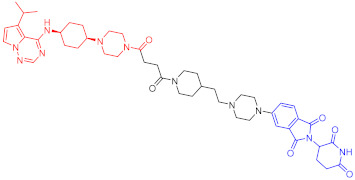	2 nM	98	[149]
46	IRAK4	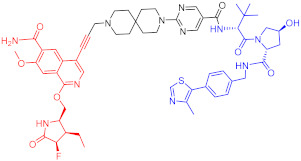	151 nM	-	[150]
47	IRAK4	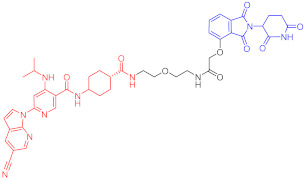	405 nM	90	[151]
48	IRAK4	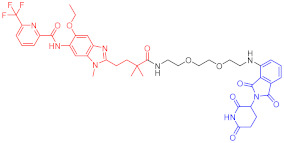	-	-	[152]
49	HDAC3	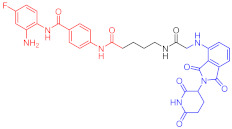	0.32μM	-	[153]
50	HDAC3	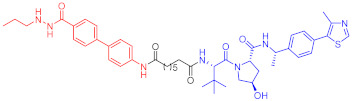	42 nM	-	[154]
51	HDAC6	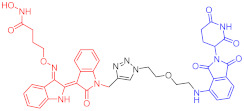	108.9 nM	88	[155]
52	H-PGDs	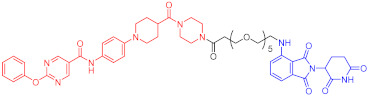	-	-	[156]
53	IDO1	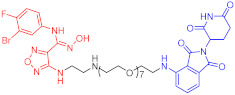	2.84 μM	93	[157]
54	Sirt2	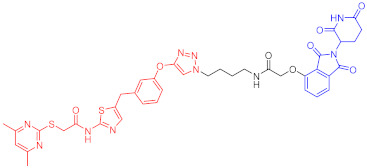	-	-	[158]
55	Sirt2	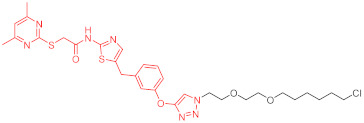	-	-	[159]
56	PCAF-GCN5	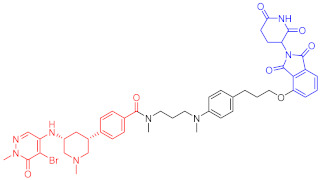	1.5–3 nM	>90	[160]
57	RIPK2	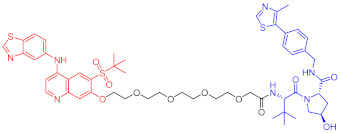	1.4 nM	>95	[36]
58	RIPK2	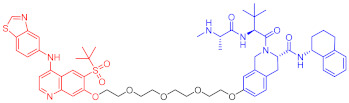	4 nM	-	[161]
59	RIPK2	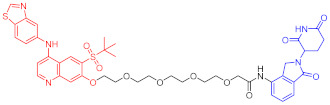	2.5 nM	-	[161]

Note: red: molecule to bind to POI, black: linker, blue: ligand of E3 ligase.

**Table 3:** Representative PROTACs for neurodegenerative diseases.

Indication	PROTAC	Target	Structure	Activity		Ref.
				DC_50_	D_max_%	
AD	60	Tau	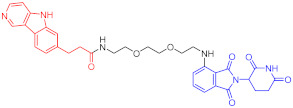	-	75	[188]
	61	Tau	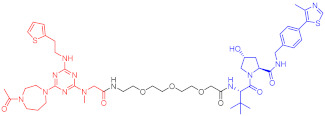	-	-	[189]
	62	GSK-3β	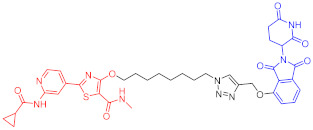	-	-	[190]
HD	63	mHtt	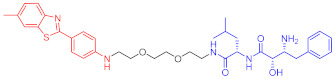	-	-	[191]
	64	mHtt	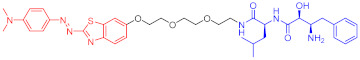	-	-	[191]
	65	mHtt	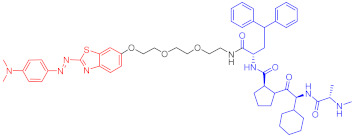	-	-	[192]
PD	66	α-synuclein	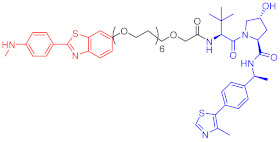	-	65	[193]

Note: red: molecule to bind to POI, black: linker, blue: ligand of E3 ligase.

**Table 4:** Representative PROTACs for cardiovascular diseases.

Protac	Target	Structure	Activity		Ref.
			DC_50_	D_max_%	
67	HMGCR	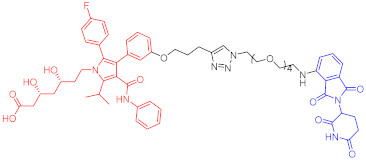	0.1 μM	-	[202]
68		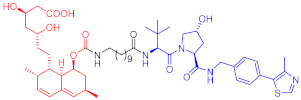	120 nM	76	[203]
69		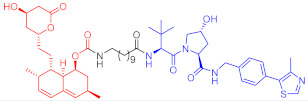	-	56	[203]

Note: red: molecule to bind to POI, black: linker, blue: ligand of E3 ligase.

**Table 5:** Representative PROTACs for antiviral.

Indication	PROTAC	Target	Structure	Activity		Ref.
				DC_50_	D_max_%	
HCV	70	NS3	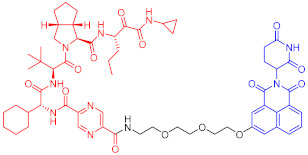	50 nM	-	[206]

Note: red: molecule to bind to POI, black: linker, blue: ligand of E3 ligase.

## Data Availability

Not applicable.
